# Anatomical, phenological and genetic aspects of the host–parasite relationship between *Andrena vaga* (Hymenoptera) and *Stylops ater* (Strepsiptera)

**DOI:** 10.1017/S0031182023000483

**Published:** 2023-07

**Authors:** Marc Hoffmann, Hanna Gardein, Henri Greil, Silvio Erler

**Affiliations:** 1 Martin-Luther-Universität Halle-Wittenberg, Halle (Saale), Germany; 2 Institute for Bee Protection, Julius Kühn Institute (JKI) – Federal Research Centre for Cultivated Plants, Braunschweig, Germany; 3 Zoological Institute, Technische Universität Braunschweig, Braunschweig, Germany

**Keywords:** genetic variability, host manipulation, host–parasite interaction, host seeking, hypermetamorphosis, life history, population, size variation

## Abstract

*Stylops ater* is an endoparasite of the mining bee *Andrena vaga* with extreme sexual dimorphism and hypermetamorphosis. Its population structure, parasitization mode, genetic diversity and impact on host morphology were examined in nesting sites in Germany to better understand this highly specialized host–parasite interaction. The shift in host emergence due to stylopization was proven to be especially strong in *A. vaga*. Around 10% of bees hosted more than 1 *Stylops*, with at maximum 4. A trend in *Stylops*' preference for hosts of their own sex and a sex-specific position of extrusion from the host abdomen was found. Invasion of *Andrena* eggs by *Stylops* primary larvae was depicted for the first time. Cephalothoraces of female *Stylops* were smaller in male and pluristylopized hosts, likely due to lower nutrient supply. The genes *H3*, *18S* and *cytochrome c oxidase subunit 1* were highly conserved, revealing near-absence of local variation within *Stylops*. Ovaries of hosts with male *Stylops* contained poorly developed eggs while those of hosts with female *Stylops* were devoid of visible eggs, which might be due to a higher protein demand of female *Stylops*. Male *Stylops*, which might have a more energy-consuming development, led to a reduction in head width of their hosts. Host masculinization was present in the leaner shape of the metabasitarsus of stylopized females and is interpreted as a by-product of manipulation of the host's endocrine system to shift its emergence. Stylopization intensified tergal hairiness, most strongly in hosts with female *Stylops*, near the point of parasite extrusion, hinting towards substance-induced host manipulation.

## Graphical abstract

**Figure:**
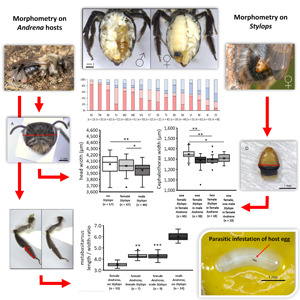

## Introduction

The strictly endoparasitic, holometabolous insect order Strepsiptera, sometimes called ‘twisted wing parasites’, comprises around 630 known species (Kathirithamby, 2018), inhabiting the entire world except for Antarctica and Greenland (Ulrich, 1929). The group is known for a number of exceptional features like extreme sexual dimorphism with larviform females (Kinzelbach, 1971), hypermetamorphosis with extremely miniaturized primary larvae (Kathirithamby, 1989; Pohl and Beutel, 2019), exceptional eyes (Buschbeck *et al*., 2003) and traumatic insemination (Peinert *et al*., 2016).

In host choice, the species *Stylops ater* Reichert, 1914 is mostly confined to *Andrena vaga* Panzer, 1799 (grey-backed mining bee), which is specialized on *Salix* (willow) pollen, often nests in large aggregations of several thousands and is relatively common in sand-rich regions in central Europe (Jůzová *et al*., 2015; Westrich, 2019). In Germany, non-parasitized bees of *A. vaga* are active between March and May (Westrich, 2019).

**Fig. 1.**
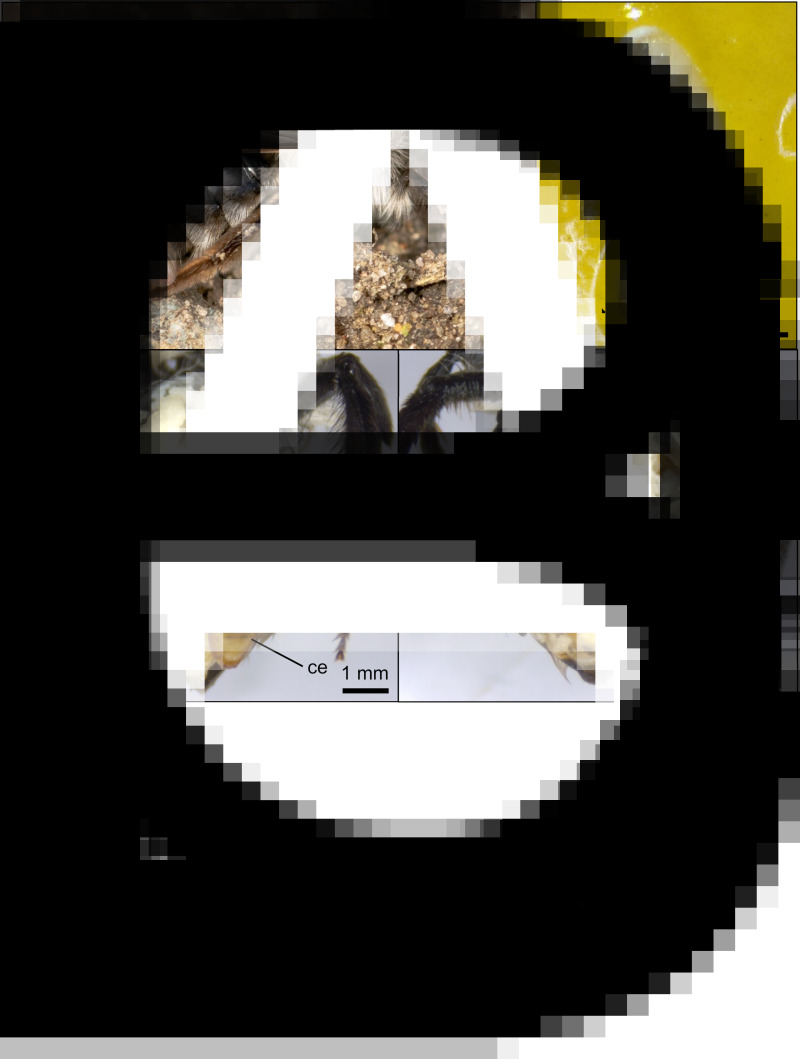
(A) Two male *Stylops ater* in copulation attempt with an extruded female (not visible in photograph) in a female *Andrena vaga* [Theaterpark (Braunschweig), 25.02.2021; photograph credit: Robin Schmidt]. (B) Egg of *A. vaga* with primary larvae of *S. ater* on pollen provision in brood cell. (C) Dissected abdomen of female *A. vaga* parasitized by female *S. ater*. (D) Dissected abdomen of female *A. vaga* formerly parasitized by male *S. ater* (ce, cephalothorax; il, ileum; mp, male puparium; re, rectum; sa, *Stylops* abdomen).

At the point of appearance of their host bee, male puparia and female cephalothoraces are already extruded from the host's abdomen, the position regularly being laterally in the intersegmental membrane between the 4th and 5th tergite and rarely between other tergites or even sternites (Linsley and MacSwain, 1957; Jones *et al*., 1980; Balzer and Davis, 2020). Shortly after host emergence, male *Stylops* emerge and are immediately able to fly while females permanently remain inside, apart from the cephalothorax, and release strong pheromones to attract mates (Cvačka *et al*., 2012; Tolasch *et al*., 2012; Löwe *et al*., 2016). *Stylops* males copulate with the extruded female parasite (Fig. 1A). Agile primary larvae with a length of around 200 *μ*m are asynchronously released in high numbers (up to 7000) after ~20–40 days through a ventral brood canal (Linsley and MacSwain, 1957; Jones *et al*., 1980; Fraulob *et al*., 2015; Balzer and Davis, 2020). They infest new hosts at an early developmental stage (Linsley and MacSwain, 1957; Knauthe *et al*., 2016) and have been shown to reach them by phoretic transport in the hair, pollen load or even inside the crop of flower-visiting bees (Ulrich, 1956; Linsley and MacSwain, 1957; Balzer and Davis, 2019).

Few studies have looked at the prevalence of stylopized bees in *Andrena* populations and further traits of parasitization. Jensen (1971) found a stylopization rate of 18% in a Danish aggregation of *A. vaga* and remains the only author to examine this specific host–parasite interaction. While the latter did not distinguish between male and female hosts and parasites, Pierce (1909) found females to be over-represented in stylopized individuals in a population of *Andrena crawfordi* Viereck, 1909. The prevalence of pluristylopization (≥2 *Stylops* per host) seems to vary greatly across host species, region and year (Jones *et al*., 1980; Balzer and Davis, 2020).

Due to the short life span of male *S. ater*, genetic exchange between populations is mainly driven by migration of infested or larva-carrying host bees (Tolasch *et al*., 2012). Aggregations of *A. vaga* are in strong genetic exchange in areas meeting the requirements of this specialized bee as shown for Northern Germany (Exeler *et al*., 2008). Therefore, a relatively high genetic homogeneity is to be expected from their parasites but was not investigated in detail so far.

Regarding the morphology of *Stylops*, Maeta *et al*. (2001) as well as Balzer and Davis (2020) found that the female cephalothorax width varied with extrusion position in related Strepsiptera taxa but the effect of host sex and the host's degree of stylopization on this feature was not examined so far.

Effects of stylopization on the host are dramatic and include changes in phenology and behaviour (Nakase and Kato, 2021) as well as internal and external morphology. *Andrena* bees parasitized by *Stylops* emerge a few weeks earlier than their conspecifics (Pierce, 1909; Brandenburg, 1953; Straka *et al*., 2011). Internal morphological alterations due to stylopization include a slight (in hosts with male *Stylops*) or strong (in hosts with female *Stylops*) ovary reduction in females (Brandenburg, 1953; Balzer and Davis, 2021), while male bees showed less alteration in external and internal copulatory organs (Salt, 1927). External morphological alterations often include the expression of characteristics of the opposite sex and therefore a phenotypical intersexualization (Pérez, 1886; Pierce, 1909; Salt, 1927; Brandenburg, 1953). Typical female features associated with pollen transport (scopa and flocculus) are reduced in stylopized females and enhanced in stylopized males, while typical male features such as the bright facial markings present in many *Andrena* species are reduced in stylopized males and enhanced in stylopized females. These alterations increase in magnitude with a growing number of *Stylops* per host and have been observed to be more severe in bees hosting male parasites (Salt, 1927). The latter also reported a slight decrease in size of the whole body and especially the males' head in stylopized individuals of some *Andrena* species. The tergal cuticula of stylopized *Andrena* bears a stronger vestiture, especially near the extrusion location of the parasite (Brandenburg, 1953; Pohl *et al*., 2020).

Considering the partially poor understanding of the biology of Strepsipterans such as *S. ater* and its simultaneous relevance due to the possible effects on populations of important pollinators, the objectives of the present study were (1) to assess parasitization of *A. vaga* by *S. ater*, including pluristylopization, parasite sex ratio and preferences for host sex and position of extrusion, (2) to gain clarity on the stage and mode of host infestation in *Stylops*, (3) to examine species diversity of the parasites and genetic variability in *S. ater* from different *A. vaga* aggregations and possible correlations of genetic and spatial distance, (4) to investigate the impact of host sex and parasitization level on female *Stylops* size and (5) to verify the impact of infestation by *S. ater* on the phenology and morphology of *A. vaga* and discuss possible implications.

## Materials and methods

### Sampling

*Andrena* host specimens were collected in 2 phases of sampling from 23rd February to 3rd March 2021 and from 12th April to 16th May 2021 (for geographic location and details of sampling sites and dates, see Fig. S1 and Tables S1 and S2). All sites comprised of 15 *A. vaga* aggregations of varying distances in and around the city of Braunschweig (Lower Saxony, Germany) as well as 2 aggregations in Göttingen (Lower Saxony) and 1 in Kassel (Hesse). Bees were collected with hand-held insect nets regardless of visible stylopization. Additionally, free-flying *Stylops* males were sampled randomly for genetic studies. The 2nd sampling was incomplete and served mainly to sample unstylopized female specimens from 12 of the sites sampled in the 1st phase, for control measurements. Unstylopized male species for comparison were obtained only from 1 site (TU – TU Nordcampus) and comprised of specimens caught in pan-traps during the annual wild bee monitoring of the BeesUp project. Specimens were sacrificed in a freezer and preserved in 96% ethanol.

A total of 25 intact brood cells were excavated from an *A. vaga* aggregation at the site MS (Melverode Sportplatz) (6 on 4th May 2022 and 19 on 11th May 2022). For that purpose, a surface area of 0.7 m^2^ was dug out to a depth of around 0.5 m, where most of the cells were found.

### Parasitization analysis and search for *Stylops* larvae

Bees, brood cells and pollen loads were analysed for parasite occurrence (Fig. 1C and D) using a Zeiss Stemi 305 binocular microscope; photographs were captured with a Zeiss Axiocam (Carl Zeiss Microscopy, Oberkochen, Germany). Female *Stylops* and males that had not yet emerged were removed and stored separately in 96% ethanol. Parasitization by already emerged male *Stylops* was detected by a hole in the intersegmental membrane and remains of the empty male puparium. Number and sex, as well as segment interval and side of *Stylops* extrusion in each bee were noted.

Bee eggs and larvae from excavated brood cells were examined from the outside and also dissected to search for parasitic larvae inside. Pollen provisions from 5 brood cells were suspended in ethanol and dispersed on a paper sheet to be gradually examined for *Stylops* primary larvae. Pollen loads of 20 representative unstylopized *A. vaga* females from the 2nd sampling period in 2021 (here: 24th April to 15th May) were examined for *Stylops* primary larvae by thoroughly shaking the bee in an ethanol-filled 2 mL tube and examining the resulting suspension in the same way as the pollen provision from the brood cells.

### DNA extraction, polymerase chain reaction (PCR) and phylogenetic analysis

A total of 127 representative *Stylops* females and 26 males were selected for genetic analysis. DNA was extracted from the cephalothorax using proteinase K (20 mg mL^−1^) digestion followed by a standard salt DNA extraction protocol (Bruford *et al*., 1992). Amplification for parts of the mitochondrial barcoding gene *cytochrome c oxidase subunit 1* (*COI*) and the nuclear genes *H3* and *18S* rRNA was realized using gene-specific primers and protocols (Tables S4–S6).

Unidirectional Sanger sequencing of PCR fragments was performed for 153 (*COI*), 21 (*H3*) and 23 (*18S*) individuals by LGC Genomics (Berlin, Germany). Sequences were manually inspected and trimmed using CodonCode Aligner (V.10.0.2, CodonCode Corporation, Centerville, MA, USA). MEGA7 (Kumar *et al*., 2016) was used for sequence alignments before blasting them for species assignment using nucleotide BLAST provided by GenBank (NCBI). MEGA7 was also used for constructing a maximum-likelihood (ML) phylogenetic tree for the gene *COI*, based on the Tamura–Nei model (Tamura and Nei, 1993) with a discrete Gamma distribution (+G) and analysis of evolutionary invariability (+I), computed with 100 ML bootstrap replications. This model was proposed as the best fit by the model finder provided in MEGA7. Voucher sequences from Smit *et al*. (2020) and Jůzová *et al*. (2015) were incorporated in the phylogenetic tree for reference and as outgroups.

### Preparation and morphological measurements

In a total of 133 representative *Stylops* females from 9 sites, the cephalothorax width as a proxy for body size was measured as shown in Fig. S2.

A representative selection of 157 *A. vaga* females and 14 males from 5 sampling sites with high numbers of sampled individuals (IW – Inselwall-Park, MS, RK – Rüningen Kirche, SD – Schillstraße Denkmal, TU), either bearing a single female/male or no *Stylops*, were chosen for morphometric examination of the head, hind legs and abdomen by using a Zeiss Stemi 305 microscope equipped with the Zeiss Axiocam camera module and ZEN Core 2 v2.5 measuring software (Carl Zeiss Microscopy, Oberkochen, Germany). Additionally, from 2 representative sites (TU, RK), the intertegular distances (ITDs) were measured. From 1 representative site (MS), female bees were dissected for the examination of their ovaries. Where possible, all measurements and examinations were conducted without knowledge on the parasitization status of the host bee to avoid bias.

Head width and ITD as 2 proxies for body size/weight (O'Neill, 1983; Hagen and Dupont, 2013) were measured (Fig. S2). For the head width, preliminary tests showed that the deviation of independent measurements was lower than 1%, so that only 1 photograph was captured per bee. For the ITDs, specimens were measured thrice and the average was used for further comparison. The head width was additionally divided by the ITD as a form of normalization to body size.

The width and length of the metabasitarsus were measured (Fig. S2) to obtain their ratio as a proxy for the shape of the whole leg. The length was additionally divided by the ITD as a form of normalization to the body size. To eliminate effects of the photographic angle, the joint was isolated and fixed on a clear plastic foil using clear tape.

Ovaries were isolated from the abdomen and one of the 3 scores (Fig. S3) was attributed to the system in Beani *et al*. (2011). The minimal egg length for an attribution of score 3 (1000 *μ*m) was derived from investigations of Brandenburg (1953).

Finally, abdomens were cleaned and dried using soapy water, acetone and a hairdryer. Hair coverage of the 4th tergite was estimated separately for the left and right half of the segment by attributing one of the 5 pre-designated scores (Fig. S5A).

### Statistical analysis

Statistical analyses were performed using standard spreadsheet software (Excel 2016). Ratios of population characterization were analysed using chi-squared (*χ*^2^) tests. Parametric data (measurements on head, thorax and legs of *Andrena* and cephalothorax of *Stylops*) were examined for normal distribution using Kolmogorov–Smirnov tests and groups were compared using analysis of variance (ANOVA) and post-hoc Student's *t*-tests. For non-parametric data (abdominal hair scores), a Kruskal–Wallis test and post-hoc Mann–Whitney *U*-tests were used. Possible correlation of *Stylops* cephalothorax width and *Andrena* head width was examined by Pearson correlation. All *P* values obtained from multiple testing were corrected using the Bonferroni–Holm method.

## Results

### Parasitization analysis and search for *Stylops* larvae

**Fig. 2.**
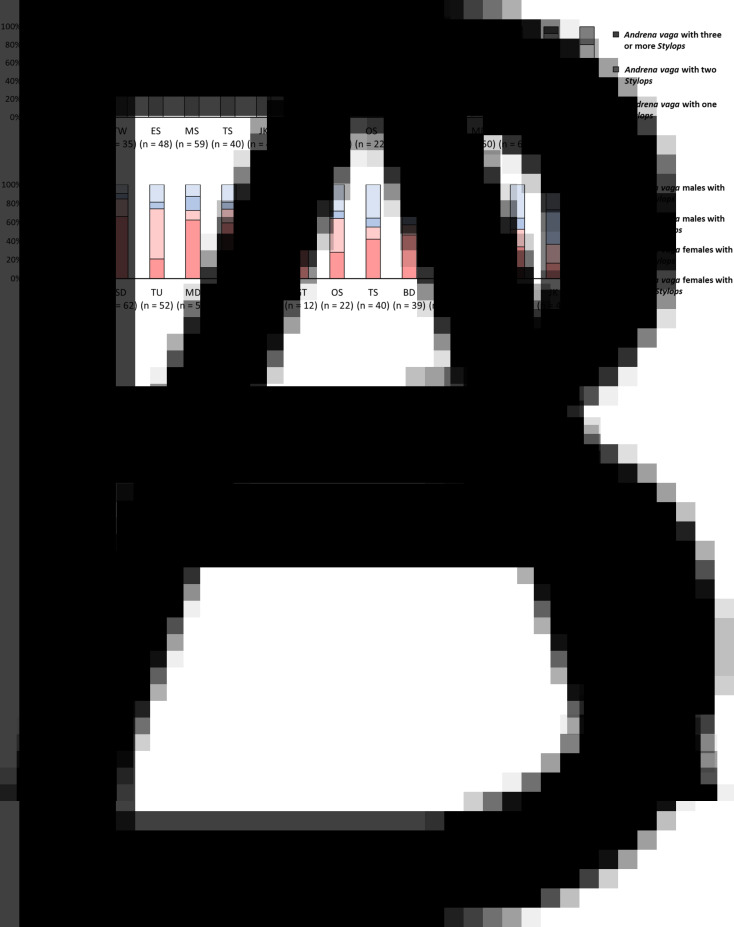
Summary of 14 *A. vaga* aggregations in Braunschweig, Göttingen and Kassel (see Table S1 for details) from the 1st sampling phase. (A) Percentage of *A. vaga* infested by different numbers of *Stylops* (different shades of grey). (B) Percentage of male and female *Stylops* in male and female *A. vaga* (different shades of red and blue).

In the 1st sampling phase, all 508 *A. vaga* individuals were stylopized without exception and hosted a total number of 574 present or emerged (male puparia) *Stylops*. The sex ratio of hosts and parasites as well as the prevalence of pluristylopized hosts among different sampling sites is shown in Fig. 2. Variability among sites was rather high but showed clear trends in host and parasite sex ratios.

On average, among the sampled stylopized individuals, females had a significantly higher share (61.6%) than males (*χ*^2^ test: *χ*^2^(1) = 26.22, *n* = 487, *P* = 0.004). The percentage of female *Stylops* was significantly higher (64.1%) in female *Andrena* (*χ*^2^(1) = 25.15, *n* = 315, *P* = 0.006) while that of male *Stylops* was significantly higher (61.7%) in male *Andrena* (*χ*^2^(1) = 10.49, *n* = 193, *P* = 0.013). Out of 249 parasitizations by male *Stylops*, only 12 were still present and the cephalotheca of their puparium externally visible.

Overall, the percentages of hosts with 1 parasite were 89.57% (454 bees), with 2 parasites was 8.86% (46 bees), with 3 parasites was 1.38% (7 bees) and only 0.2% (1 bee) were infested with 4 *Stylops.* The prevalence of pluristylopization was slightly higher in female hosts (10.84%) than in male hosts (9.73%) and slightly higher among female *Stylops* (7.77%) than among male *Stylops* (5.08%). Of the 46 bees infested by 2 *Stylops*, 10 hosted 2 males, 16 hosted 2 females and 20 hosted a male and a female.

The position of extrusion was usually the intersegmental membrane between the 4th and 5th abdominal tergite, only 15 (2.6%) of the *Stylops* extruded from between the 3rd and 4th tergite. In that case, apart from 1 female and 1 male *Stylops*, the host was always pluristylopized. Regarding the side of extrusion (left or right) of single *Stylops* from the abdomen of their host, there was a general tendency for females to extrude from the right half of the abdomen (57.20%) and for males to extrude from the left half of the abdomen (61.31%), which was significant only in the latter case (*χ*^2^(1) = 10.18, *n* = 199, *P* = 0.014). Only 8 *Stylops* emerged from the middle of the tergite intersegmental membrane. Of these, 6 developed in hosts with at least 3 parasites and were therefore displaced from the usual side position.

In the 2nd sample phase, only 2 of 150 *A. vaga* were infested, both with a female *Stylops.* These *Stylops* were strongly enlarged and contained eggs and weakly pigmented embryos in high quantities.

Of the 19 excavated brood cells, presumably of *A. vaga*, 7 contained recently hatched *Andrena* larvae of ~3–4 mm. Two cells contained a different kind of bee larvae that were tentatively assigned to *Nomada lathburiana* Kirby, 1802, a specialized cleptoparasite of *A. vaga*. The other 10 cells contained elongated eggs of ~2.5 mm length. In 4 of them, dark primary larvae (or their exuviae) of ~200 *μ*m length were visible (Fig. 1B; 3 contained 1 primary larva each, 1 contained 3 primary larvae). A closer examination showed that they all were situated in the gap between the chorion and the developing embryo. While most *Andrena* larvae were alive, the *Stylops* larvae found in the eggs were motionless. Further dissection of bee eggs and larvae did not reveal any more *Stylops* larvae.

Five examined pollen provisions (3 from cells containing stylopized eggs and 2 additional ones) did not contain any *Stylops* primary larvae, nor did any of the 20 investigated pollen loads of provisioning *A. vaga* females.

### Phylogenetic analysis

**Fig. 3.**
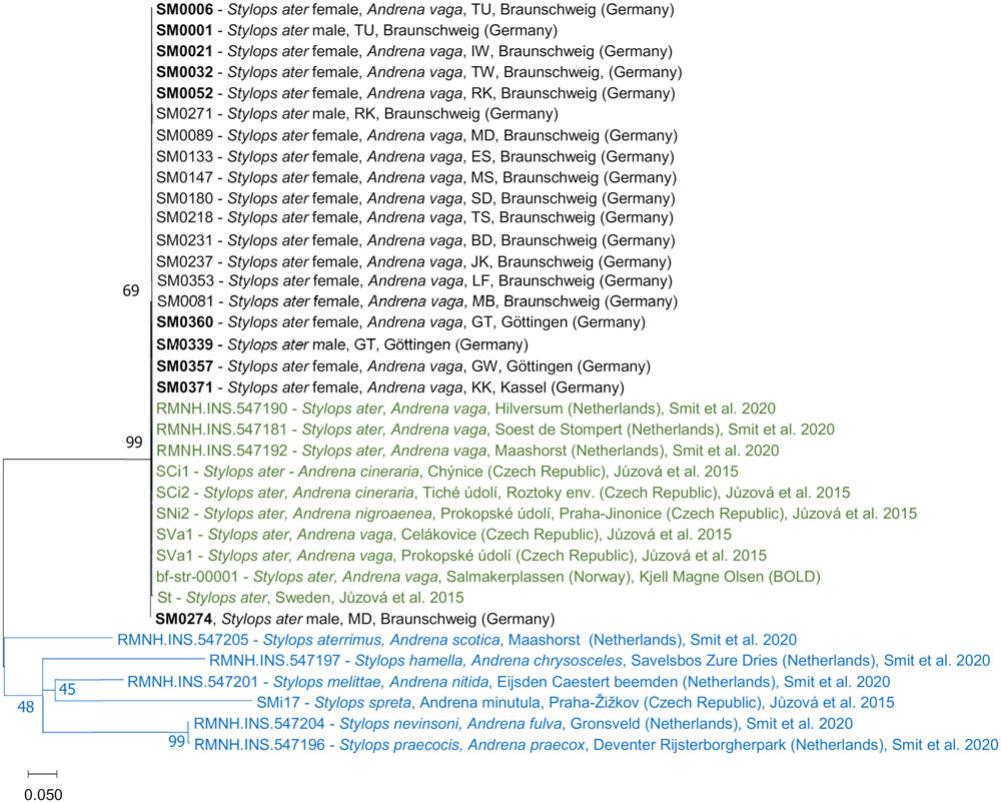
ML phylogenetic tree for the gene *COI* of selected *Stylops* specimens. Based on the Tamura–Nei model with a discrete Gamma distribution [5 categories (+G, parameter = 0.934)] and analysis of evolutionary invariability [(+I), 62.29% sites], computed with 100 bootstraps, with a total of 535 positions in the final dataset. Branch lengths are measured in the number of substitutions per site. Labels show *Stylops* species and sex (if known), host species, site, country and (if applicable) source of sequences. Database-obtained samples of *S. ater* are shown in green; database-obtained samples of other *Stylops* species that serve as an outgroup are shown in blue. Voucher sequences uploaded to NCBI GenBank are in bold.

Blasting of *COI* sequences revealed a genetic identification as *S. ater* for all 127 sequenced female *Stylops* from *A. vaga* and 26 free-flying males. Therefore, it can be assumed that all consequences for the bees shown in this study were caused by this single parasite species. Sequences for *COI*, *H3* and *18S* were extremely conserved among individuals. Species unity and very high conservation can also be seen in the ML phylogenetic tree for *COI*, including earlier voucher sequences for *S. ater* and *Stylops* spp. (Fig. 3). Sequences of representative specimens are accessible from GenBank (NCBI) under their IDs (OQ333011–OQ333020).

Only in 1 male *S. ater* from the site MD (Madamenweg Discgolfanlage, voucher ID: SM0274), a single base transition (G–A) was found in the *COI* sequence at position 292. Another transition (C–T) was found in the *COI* sequence (position 276) of an *S. ater* from Sweden (sample ID: st) from Jůzová *et al*. (2015).

### Size variation of *Stylops* females

**Fig. 4.**
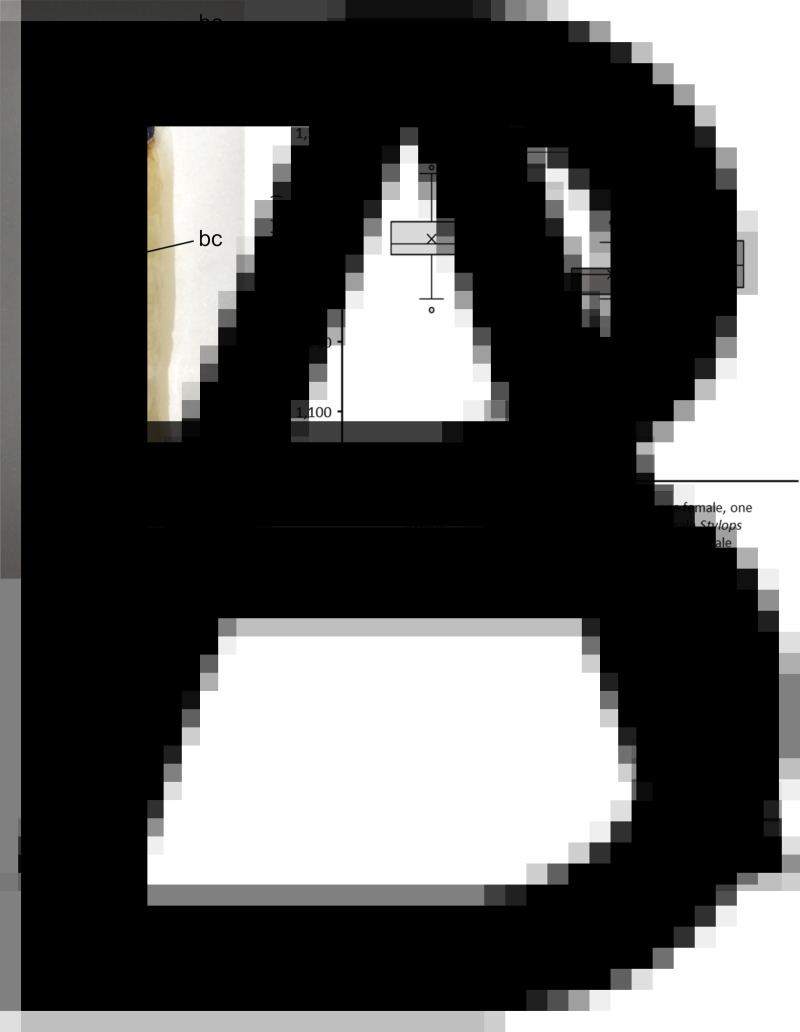
Cephalothorax width variation in *S. ater* females. (A) Female extracted from the host abdomen (ce, cephalothorax; bo, birth opening; bc, brood canal). (B) Cephalothorax width of *Stylops* females in different host–parasite situations. Boxplots show minima and maxima (whiskers), medians and 1st and 3rd quartiles (boxes), means (cross) and outliers (empty circles) (*P < 0.05, **P < 0.01).

Cephalothorax widths of *Stylops* females from monostylopized male and female hosts were normally distributed (Kolmogorov–Smirnov test: *n* = 65, *Z* = 0.11, *P* = 0.385) and no site effect was found in male or female hosts (ANOVA: d.f._female_ = 64, *P*_female_ = 0.568, d.f._male_ = 33, *P*_male_ = 0.319). Consequently, *Stylops* from rarer parasitization situations (2 females, 1 female and 1 male) were pooled among sites. The average cephalothorax width of *Stylops* females was significantly higher (5.22%) in monostylopized female *A. vaga* hosts compared to monostylopized male hosts [Student's *t*-test: *t*(48) = 4.13, *P* = 0.002] (Fig. 4). The *Stylops*' cephalothorax was significantly narrower when another female *Stylops* was present in the female host [3.93%, *t*(81) = 4.31, *P* = 0.002] or when it previously shared the host with a male *Stylops* [2.97%, *t*(73) = 2.64, *P* = 0.042]. The sex of the 2nd parasite had no effect on cephalothorax width [*t*(26) = −0.66, *P* = 0.501]. There was no significant correlation between the head width of monostylopized female host bees and the cephalothorax width of their parasite (Pearson correlation, *n* = 46, *r*^2^ = 0.055, *P* = 0.718).

### Impact of stylopization on host morphology

In all dissected unstylopized *A. vaga* females (*n* = 10), ovaries were normally developed and contained small as well as fully developed eggs (score 3, Fig. S3). Bees infested by a single female *Stylops* (*n* = 10) showed no obvious egg development in their tube-like ovaries, which were not reduced in length (score 1, Fig. S3). The ovaries of bees infested by a single male *Stylops* (*n* = 10) contained small, poorly developed eggs (score 2, Fig. S3).

Average ITDs of *A. vaga* females were normally distributed (*n* = 55, *Z* = 0.121, *P* = 0.368). There was no significant difference between infested and uninfested bees [ANOVA: *F*(5) = 1.610, *P* = 0.175] (Fig. S4A).

**Fig. 5.**
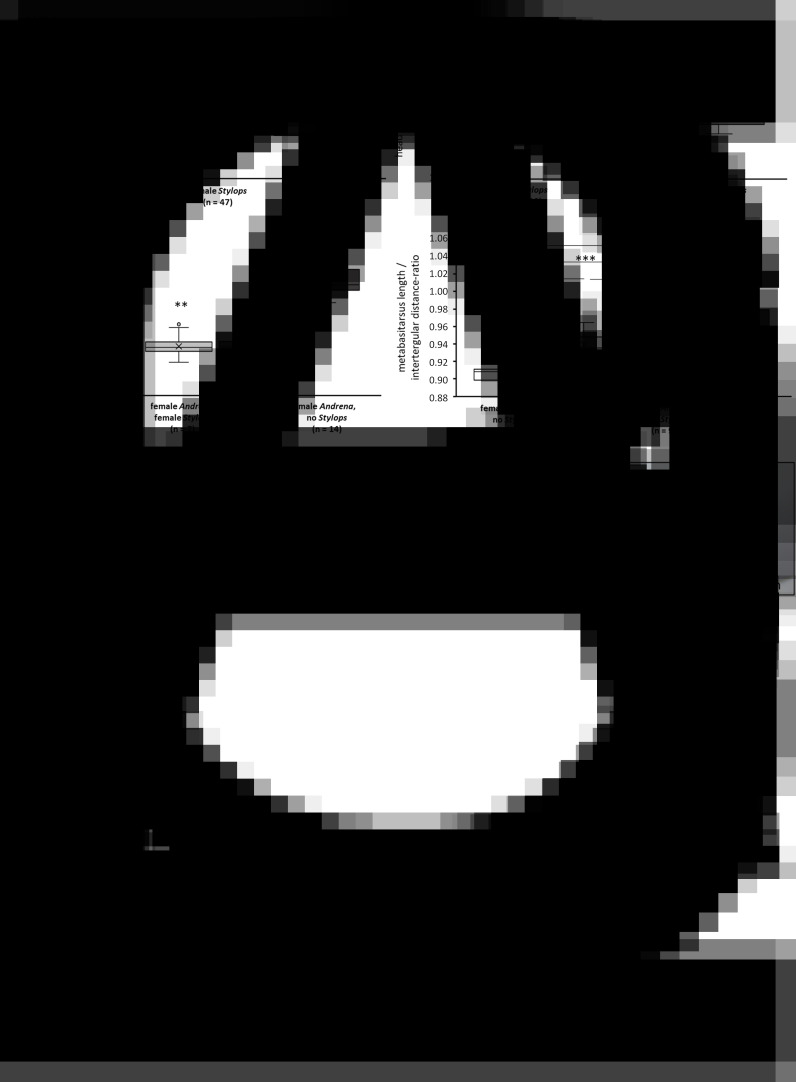
Summary of host bee morphological changes. (A) Head width of *A. vaga* females of different stylopization status. (B) Head width/ITD ratio of *A. vaga* females of different stylopization status (site TU). (C) Metabasitarsus length/width ratio of *A. vaga* of different groups. (D) Metabasitarsus length/ITD ratio of *A. vaga* of different groups. Boxplots show minima and maxima (whiskers), medians and 1st and 3rd quartiles (boxes), means (cross) and outliers (empty circles) (*P < 0.05, **P < 0.01, ***P < 0.001). (E) Hind legs of different *A. vaga* individuals (I, unstylopized female; II, stylopized female; III, unstylopized male; co, coxa; fe, femur; fl, flocculus; ml, metabasitarus length; mw, metabasitarsus width; sc, scopa; ti, tibia; tr, trochanter).

Head widths of unstylopized bees were normally distributed (*n* = 150, *Z* = 0.084, *P* = 0.231) but at 2 sites (MS, SD) differed significantly from one another in unstylopized *A. vaga* females [ANOVA and post-hoc Student's *t*-tests: *t*(22) = 3.40, *P* = 0.031]. With all sites pooled, head width was significantly reduced (by around 2.39%) in bees with a male *Stylops* compared to unstylopized individuals [*t*(101) = 3.56, *P* = 0.001] (Fig. 5A). Looking at the groups of stylopization across sites, a low mean head width of bees without *Stylops* and high mean head width of bees with female *Stylops* was noticeable for the site MS. Therefore, this site was defined as an outlier and removed from the pool for an alternative model shown in Fig. S4B, where the difference in head width became clearer and even female *Stylops* reduced their hosts’ head width significantly. For comparison, average head width of unstylopized males at the site TU was clearly lower but also more variable (3774.87 ± 222.25 *μ*m, *n* = 14).

Average head width/ITD ratio was significantly higher in bees with a female *Stylops* compared to bees without a *Stylops* [*t*(15) = 3.63, *P* = 0.019] (Fig. 5B). This means their heads, even though tending to be smaller in absolute numbers, were proportionally larger than that in unstylopized females. In a greater extent, this was also the case in unstylopized *A. vaga* males, where this ratio averaged to 1.856 ± 0.139 (*n* = 14).

In stylopized *A. vaga* females, compared to unstylopized individuals, all hind leg joints appeared more slender and the basitarsus was less tapered apically. The hair, especially the flocculus on the trochanter and the scopa on the tibia, but also on the basitarsus, were shorter and less dense. While its colour seems slightly darkened on the coxa, trochanter and femur, it appears partly brightened on the tibia and basitarsus. In all the above characteristics, the leg looks like a transitional form between that of unparasitized females and males (Fig. 5E). The metabasitarsus lengths and widths of unstylopized bees were normally distributed (*n* = 50, *Z*_length_ = 0.113, *Z*_width_ = 0.0801, *P*_length_ = 0.505, *P*_width_ = 0.880). Sites were pooled as the ratios did not differ significantly among unstylopized bees [ANOVA: *F*(4) = 1.597, *P* = 0.194]. In comparison to unstylopized females, the ratio was significantly higher in bees with a female *Stylops* [*t*(15) = 19.48, *P* = 0.002] as well as bees with a male *Stylops* [*t*(18) = 19.80, *P* < 0.001] (Fig. 5C). The latter 2 did not differ from each other [*t*(15) = 0.02, *P* = 0.417]. This means that the legs of stylopized *A. vaga* females are 16.5% leaner compared to those of unstylopized individuals. With this, they direct towards the higher ratio in *A. vaga* males (6.013 ± 0.066, *n* = 14). The metabasitarsus length/ITD ratio, in comparison to unstylopized females, was significantly higher in bees with a female *Stylops* [*t*(15) = 5.39, *P* = 0.002] and with a male *Stylops* [*t*(17) = 7.14, *P* < 0.001] (Fig. 5D). The 2 latter also differed significantly from each other [*t*(14) = 2.43, *P* = 0.045]. The ratio in bees with a male *Stylops* did not differ from that in *A. vaga* males [0.961 ± 0.133, *t*(21) = 0.2, *P* = 0.828].

A Kruskal–Wallis test and post-hoc Mann–Whitney *U*-tests revealed a significantly lower mean (left and right side) hair score for unstylopized *A. vaga* from the site MS compared to several other sites. Again, MS was excluded from further tests to avoid possible site effects. Currently, we have no explanation for the observed variance at site MS, except for the late sampling. The difference in hairiness of the 4th tergite is shown in Fig. S5. On the half where the *Stylops* was extruded, in comparison to unstylopized individuals, hair scores were generally higher in bees infested with a *Stylops* of any sex; the same was obvious for the opposite side of the segment. The difference between the 2 halves of the segment was significant (multiple Mann–Whitney *U*-tests, all *P* < 0.001) (Fig. S5). *Stylops* females had a significantly higher impact on the abdominal hairiness compared to males, both on the side of extrusion (*U* = 247, *P* < 0.001) as well as on the opposite side (*U* = 247, *P* = 0.004).

## Discussion

### Population characteristics

The rate of 100% stylopization in the 1st sampling phase is similar to the high rates found by Straka *et al*. (2011) early in the season, but in comparison to the rate found by the latter, also takes into account male *A. vaga.* The phenomenon of early emergence triggered by stylopization seems to be stronger in this species than in other *Andrena* species such as *Andrena minutula* and *Andrena milwaukeensis*, where according to Straka *et al*. (2011) and Balzer and Davis (2020), stylopized females emerge at a similar time as unstylopized males. The general advantages of synchronized, early nest emergence for the *Stylops* are firstly a higher chance for males to find a receptive female within their very short lifetime and secondly more time for the embryogenesis of the larvae that immediately need to reach a host in a recently constructed *Andrena* brood cell after emerging from their mother (Kinzelbach, 1978; Straka *et al*., 2011).

The slightly higher prevalence of females among stylopized *A. vaga* that was found here is congruent with findings of Pierce (1909). If this is not an effect of the sampling period (stylopized males might emerge later than stylopized females), a possible explanation might be that male hosts die more often due to stylopization during hibernation because of their smaller size as suggested by Straka *et al*. (2011). The current study, however, found that male *A. vaga* more often host male *Stylops* while at the same time, the negative impact on bee head width tended to be stronger with male *Stylops* at least in female hosts.

The apparent preference of female *Stylops* for female hosts and preference of male *Stylops* for male hosts seems advantageous for the parasite. This might be explained by the fact that *Andrena* females live longer than males (Westrich, 2019) and therefore allow *Stylops* females a longer time for offspring production while for male *Stylops*, which emerge right away, the longevity of their host is less crucial. Additionally, an infestation of a single host with both female and male *Stylops* may be unfavourable for the female *Stylops* as the host might die from desiccation or secondary infections after male emergence which leaves behind an open wound in the bee's abdomen (Beani *et al*., 2011). Another advantage is the presumably higher egg production possible for *Stylops* females in heavier female hosts. However, looking at the minute size and low probability of host-finding connected to phoretic transport of the *Stylops* primary larvae, it seems unlikely for them to refuse invading an egg of the unpreferred sex even if recognition of the latter seems possible due to the presence of olfactory pits (Pohl and Beutel, 2019). Brandenburg (1953) doubted that female *Stylops* are even able to reproduce at all when parasitizing *Andrena* males. However, since even females of *A. vaga* are reported to live only for ~2 weeks in the wild (Paxton, 1991; Straka *et al*., 2014), the duration of embryogenesis in *S. ater* and other central European *Stylops* species must be distinctly shorter than that found in the investigated American species (Linsley and MacSwain, 1957; Balzer and Davis, 2020), unless *Stylops* can prolong the lifespan of their hosts similar to other Strepsiptera genera (Kathirithamby, 1977; Beani *et al*., 2021). A finding seemingly contradicting the latter is the near absence of stylopized *A. vaga* even on the 1st day of the 2nd sampling phase in the current study, which might also be a result of an aberrant daytime behaviour of stylopized bees later in the season as described by Ulrich (1956).

The rate of around 10% pluristylopization is approximately twice as high as the one found for *A. milwaukeensis* by Balzer and Davis (2020) who only recorded female *Stylops*. This rate is most likely positively correlated with the general prevalence of *Stylops* in their host population and thereby subject to periodical fluctuations as found by Jones *et al*. (1980).

In the current study, the strongly prevailing extrusion position between the 4th and 5th tergite was mostly only deviated from when several *Stylops* had to share the space in the host's abdomen and displaced each other from favourably extrusion positions. In the case of triple stylopization, apart from 2 bees, the 3rd *Stylops* was always situated in the middle in a space-saving way, supported by the other 2 like Balzer and Davis (2020) documented. The observed slight preference of each *Stylops* sex for 1 side of the abdomen to extrude from is surprising and was not found by Balzer and Davis (2020) who had a smaller sample size. Since all organs in the bee abdomen are pairwise or centrally located (Carreck *et al*., 2013), this preference of *Stylops* for 1 side can only be caused by a position of the midgut that could be laterally shifted by default or must have other non-organic reasons.

The results from this population characterization confirmed the peculiarity of *S. ater* within its genus regarding host-manipulation and raised new questions about reasons for preferences in host sex and parasitization position.

### Insights into infestation mode

The observation of *Stylops* primary larvae invading a host egg is remarkable and has only been reported for an American *Stylops* species parasitizing *Panurginus melanocephalus* Cockerell, 1926 (Linsley and MacSwain, 1957). The same behaviour was also documented for the Strepsiptera genus *Pseudoxenos* in the egg of a solitary wasp by Maeta *et al*. (2001). This confirms the hypothesized morphological adaptation of the head of *Stylops* primary larvae to egg adhesion and penetration (Pohl and Beutel, 2019). Given that live host larvae were found in cells, it seems unlikely that the motionless *Stylops* might have died, from stress caused by excavation. They might have died from the exhaustion in penetrating the chorion as Maeta *et al*. (2001) observed in *Pseudoxenos*, were actually alive and only resting before their 1st moult like the *Xenos* primary larvae documented by Manfredini *et al*. (2007). Alternatively, only the 1st instar's exuviae were observed as by Linsley and MacSwain (1957) and the secondary larvae were not found inside the eggs due to their small size and pale coloration similar to the host embryo.

The lack of further *Stylops* primary larvae in the pollen provision of brood cells containing stylopized *A. vaga* eggs is surprising and indicates a smaller risk of dying for the minute primary larvae in the final step of host-seeking within the brood cell than previously expected. The complete absence of primary larvae in pollen loads of unstylopized *A. vaga* is surprising, considering the high prevalence of *S. ater* at the sampling sites and the high number of larvae produced by female *Stylops* (Jones *et al*., 1980). *Stylops* primary larvae were reported to be released asynchronously (Balzer and Davis, 2021) and in the current study, pollen provisions were collected within a period of around 3 weeks where primary larvae were found inside mature host eggs. Still, it is possible that the peak of prevalence of *Stylops* primary larvae on blossoms visited by foraging bees was missed. Another possible explanation is that the primary larvae mostly travelled within the bee's crop as Linsley and MacSwain (1957) observed and not in the hair.

By examining the brood cells of *A. vaga*, the peculiar host invasion mode of *S. ater* primary larvae was confirmed and documented for the first time. Regarding the mode of phoresy for this host–parasite system, at least travelling of numerous primary larvae in the pollen load of provisioning bees within the period of host invasion could not be confirmed.

### High conservation of considered genes

The high uniformity of sequences in *COI*, *H3* and *18S*, even between populations from different cities and countries, is congruent with the findings of Jůzová *et al*. (2015) and represents a high conservation of the genes and/or high genetic exchange between *A. vaga* aggregations (Exeler *et al*., 2008). The single-base substitution found in the *S. ater* male from the site MD has to be interpreted as a spontaneous mutation. The high variability of *COI* in the Strepsipteran *Xenos vesparum*, even within 1 host nest (Vannini *et al*., 2008), opposed to the homogeneity in the examined *S. ater*, is remarkable. The proposed bivoltine life cycle of *X*. *vesparum* (Manfredini *et al*., 2007) and the resulting shorter generation time might play a role here (Thomas *et al*., 2010).

The examination of genetic variability in *S. ater* confirms results from studies in other countries and herein endorses that relatively closely related species with different ecology can have different levels of genetic variability within 1 gene locus.

### Size variation of *Stylops* females

The comparatively lower cephalothorax width of *Stylops* females in male *A. vaga* is most likely due to the smaller weight of the host, which is in turn caused by a smaller brood cell provision (Danforth, 1990). The decrease in cephalothorax width in *Stylops* females in bistylopized hosts might be the consequence of intra-host competition for nutrients between the 2 parasites. Unexpectedly, female *Stylops* cephalothoraxes are not significantly narrower co-parasitizing with a male *Stylops* compared to a 2nd female, even though the presumably higher energy consumption of males has been shown here as well as in Beani *et al*. (2011). However, if the total egg production is positively correlated with body size/cephalothorax width as in other insects (Jervis and Ferns, 2004), reproductive success of female *Stylops* decreases in male or pluristylopized hosts.

The effect of host size and sex on *Stylops* size, which had rarely been mentioned in earlier studies, was proven here and might play a role in preferences in host-seeking due to effects on the parasite's fitness.

### Impact of stylopization on host morphology

The documented ovary underdevelopment of 2 different degrees is in line with the findings of Brandenburg (1953), even though the unstylopized bees examined here are arguably older than the stylopized ones and their ovaries and eggs had more time to develop. The latter hypothesized the stronger ovary suppression in bees with female *Stylops* to be a result of a higher protein demand for egg production. Brandenburg (1953) observed that the eggs in ovaries of bees with male *Stylops*, larger at emergence already, continued growth after the parasite emerges and concluded that the concerned bees can most likely reproduce. Therefore, female stylopization might more fatal in terms of fitness for female hosts than male stylopization. For male hosts it is the other way around: they seem to stay fertile (Salt, 1927) despite stylopization but the exit wound resulting from male stylopization likely poses a risk of infection and desiccation. The ovary reduction is linked to a decrease in size of the juvenile hormone-producing corpora allata as Brandenburg (1953) and Strambi *et al*. (1973) showed experimentally.

The trend for a reduced head width in stylopized *A. vaga* females compared to unstylopized ones may represent a loss of nutrients during development. That this effect was stronger in bees with male *Stylops* is congruent with the findings of Beani *et al*. (2011) who showed that in *Polistes*, the lipid stores of wasps hosting male *Xenos* were smaller than in those hosting females. A plausible reason might be the higher energy demand for complete metamorphosis of male Strepsiptera compared to the larviform females (Lease and Wolf, 2011). The observed higher head width/ITD ratio in *A. vaga* with female *Stylops* could be interpreted as a 1st masculinized feature, even though it is not as obvious as the following one. However, it is contrary to the findings of Salt (1927) who reported the heads of some stylopized *Andrena salictaria* Robertson, 1905 to be ‘ridiculously small’ but also stated that this effect is only measurable in male bees.

The hind legs of stylopized females are clearly overall masculinized in shape and size in relation to their body. A stronger impact of male *Stylops* in this aspect, as reported in Pierce (1909), was not found. Since at least *A. vaga* females with a male *Stylops* are suspected to be able to reproduce after the emergence of their parasite, this permanent alteration of the shape and vestiture of their legs, which are associated with provisioning their offspring with pollen, may lead to a further decrease of their reproductive fitness.

The denser and longer tergal hair of stylopized *A. vaga* is very conspicuous and even visible on photographs. The fact that this effect was strongest near the cephalothorax of female *Stylops* might be a hint towards the masculinization being a substance-induced host manipulation instead of a mere by-product of nutrient depletion or spatial gonad restriction. Whether it is only a side effect of other aspects of host manipulation or is of advantage for *Stylops* remains unclear. Pohl *et al*. (2020) tested the hypothesis that it represents a parasitic manipulation that facilitates adhesion of the male *Stylops* to the host's body during copulation but did not find a difference in traction force of male tarsi to smooth and hairy surfaces. They suggested that the increase in hairiness could still be a parasitic manipulation that facilitates phoretic transport of the *Stylops* primary larvae to flowers.

By examining different traits of stylopized *A. vaga* females and comparing them to unstylopized individuals, morphological alterations of different suspected origins were found. It seems plausible that the main aim of *Stylops* manipulation of their *Andrena* host is a shift in emergence to gain time for embryogenesis by exploitation of the pre-existing protandry in this genus and that all morphological alterations are merely a by-product (with a potential advantage for the parasite) of their impact on the host's endocrine system (Strambi *et al*., 1973; Straka *et al*., 2011).

To resolve some of the addressed issues concerning population structure and host manipulation in *S. ater* and its host *A. vaga*, future studies should perform a different type of sampling that reduces various sources of bias by using emergence traps over a longer time span. Host marking experiments and successive examination of *Stylops* egg development may answer questions whether female parasites have a life-prolonging effect on its host, whether male bees live long enough to ensure offspring dispersal for *Stylops* females, and whether the lifespan of bees formerly hosting a male *Stylops* is reduced due to emergence wounding. Biochemical studies focusing on hormone levels of the developmental stages of the host and its parasite might shed light on the exact ways of host manipulation that lead to all of the before mentioned effects and thereby add to a better understanding of complex host–parasite interactions.

## Supplementary material

For supplementary material accompanying this paper visit https://doi.org/10.1017/S0031182023000483. click here to view supplementary material
