# Supplementary material for: Anatomical, phenological and genetic aspects of the host–parasite relationship between Andrena vaga (Hymenoptera) and Stylops ater (Strepsiptera)
Source: Parasitology. 2023 May 9;150(8):744–53. doi: 10.1017/S0031182023000483 (PMC10410535; doi:10.1017/S0031182023000483)
Supplement: Supplementary file 1 [file S0031182023000483sup001.pdf]

Supplementary material:

Anatomical, phenological and genetic aspects of the host-parasite relationship between *Andrena vaga* (Hymenoptera) and *Stylops ater* (Strepsiptera)

Marc Hoffmann[1,2], Hanna Gardein[2], Henri Greil[2], Silvio Erler[2,3,*]

[1]Martin-Luther-Universität Halle-Wittenberg, Halle (Saale), Germany; [2]Institute for Bee Protection, Julius Kühn Institute (JKI) - Federal Research Centre for Cultivated Plants, Braunschweig, Germany; [3]Zoological Institute, Technische Universität Braunschweig, Braunschweig, Germany

* **Authors for correspondence:** Silvio Erler, E-mail: silvio.erler@julius-kuehn.de; Marc Hoffmann, E-mail: hoffmann.marc.contact@freenet.de

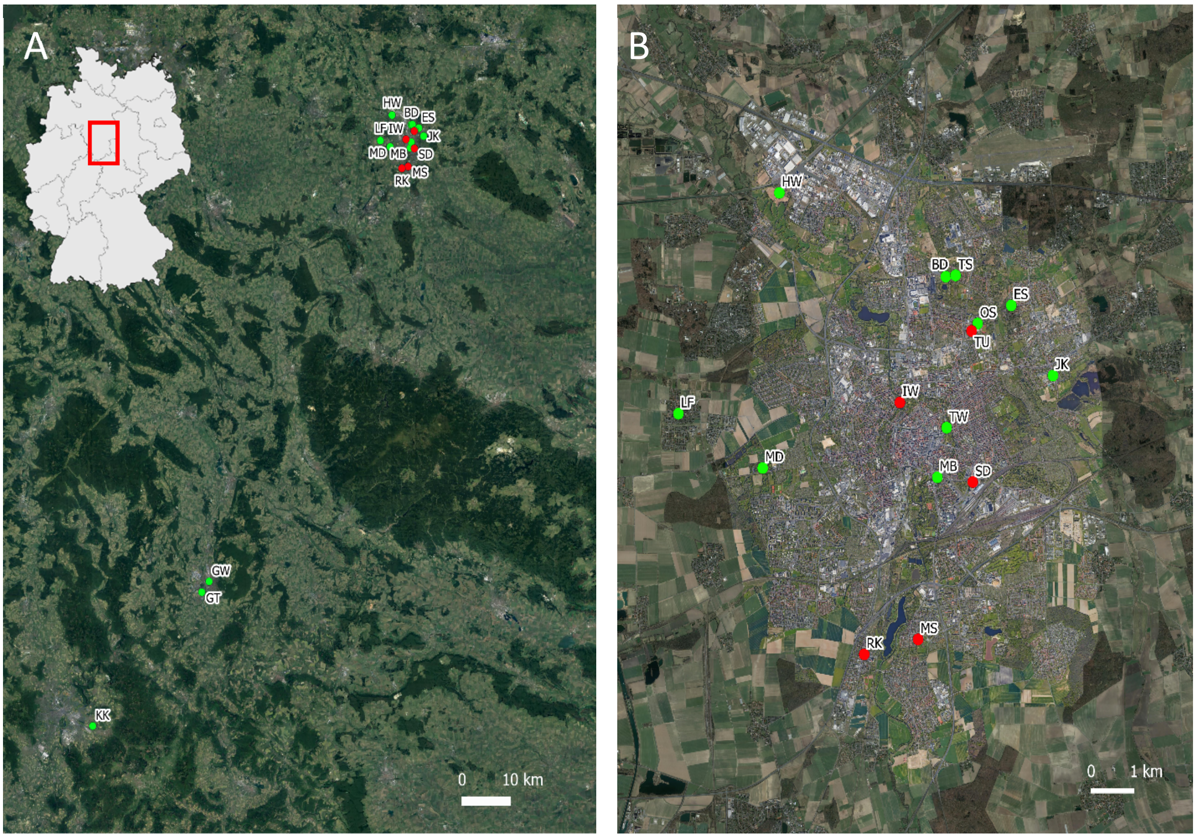

Figure S1: Maps of the study sites. (A) Location in Germany and spatial relation between sites in Braunschweig, Göttingen and Kassel. (B) spatial relation between sites in Braunschweig. Red dots show sites with high sample sizes that were selected for morphometrical analysis. Ground map: Google Maps (2021) Available at: https://www.google.com/maps (Accessed: 25th May 2022) Map crated using *QGIS 3.4 'Madeira'*

Table S1: Name and location of sites for *Andrena vaga* sampling.

Site code	Site name	Site city	Coordinates (lat, lon)
BD	Bahnübergang Dowesee	Braunschweig	52.295526, 10.531960
ES	Essener Straße	Braunschweig	52.290064, 10.554394
GT	Göttingen Tonkuhlen	Göttingen	51.518852, 9.910113
GW	Göttingen Stadtwall	Göttingen	51.536757, 9.931576
HW	Hafen Weggabelung	Braunschweig	52.311458, 10.474944
IW	Inselwall-Park	Braunschweig	52.271593, 10.516237
JK	Julius Kühn-Institut	Braunschweig	52.276727, 10.568632
KK	Kalkberg Kassel	Kassel	51.294123, 9.586104
LF	Lamme Friedhof	Braunschweig	52.269460, 10.440353
MB	Mühlbergweg	Braunschweig	52.257347, 10.529043
MD	Madamenweg Discgolfanlage	Braunschweig	52.259126, 10.469246
MS	Melverode Sportplatz	Braunschweig	52.226508, 10.522466
OS	Ottenroder Straße	Braunschweig	52.286607, 10.542855
RK	Rüningen Kirche	Braunschweig	52.223645, 10.504000
SD	Schillstraße Denkmal	Braunschweig	52.256453, 10.541196
TS	Theisenstraße	Braunschweig	52.295768, 10.535313
TU	TU Nordcampus	Braunschweig	52.285188, 10.540780
TW	Theaterwall-Park	Braunschweig	52.266801, 10.532239

Table S2: Dates of *Andrena vaga* sampling.

Site	Sampling date	Bees collected	Collector(s)
JK	23.02.2021	26	Marc Hoffmann
BD	24.02.2021	7	Jana Deierling
HW	24.02.2021	4	Jana Deierling
IW	24.02.2021	29	Marc Hoffmann
SD	24.02.2021	47	Harmen Hendriksma
MB	24.02.2021	10	Harmen Hendriksma
TW	24.02.2021	21	Marc Hoffmann
BD	25.02.2021	14	Jana Deierling
LF	25.02.2021	11	Dennis Leer
MB	25.02.2021	7	Henri Greil and Harmen Hendriksma
MD	25.02.2021	25	Dennis Leer
MS	25.02.2021	46	Henri Greil and Harmen Hendriksma
OS	25.02.2021	12	Dennis Leer and Jana Deierling
RK	25.02.2021	55	Henri Greil and Harmen Hendriksma
TS	25.02.2021	20	Jana Deierling
TU	25.02.2021	23	Dennis Leer and Jana Deierling
JK	02.03.2021	3	Jana Deierling
LF	02.03.2021	4	Marc Hoffmann
MD	02.03.2021	14	Marc Hoffmann
BD	03.03.2021	5	Jana Deierling
ES	03.03.2021	38	Henri Greil
GT	03.03.2021	12	Felix Klaus
GW	03.03.2021	3	Felix Klaus
KK	03.03.2021	23	Ira Waldow
MB	03.03.2021	10	Marc Hoffmann
OS	03.03.2021	10	Marc Hoffmann
TS	03.03.2021	9	Jana Deierling
TU	03.03.2021	19	Marc Hoffmann
JK	12.04.2021	14	Marc Hoffmann
BD	24.04.2021	13	Hanna Gardein
IW	24.04.2021	15	Marc Hoffmann
SD	24.04.2021	15	Marc Hoffmann
MB	24.04.2021	14	Hanna Gardein
TS	24.04.2021	11	Hanna Gardein
TW	24.04.2021	14	Marc Hoffmann
RK	25.04.2021	10	Hanna Gardein
ES	09.05.2021	10	Hanna Gardein
TU	09.05.2021	10	Hanna Gardein
MS	15.05.2021	13	Hanna Gardein
MD	16.05.2021	11	Hanna Gardein

Table S3: Protocol for DNA extraction from a single *Stylops* sample.

Step	Action
**1**	Crushed tissue (cephalothorax of females, whole body of males) + 410 µL extraction buffer (Tris 0.01 M, NaCl 0.1 M, EDTA 0.01 M, ddH_2O) + 80 µL SDS (10%) + 20 µL Proteinase K (20 mg/ml)
**2**	incubate shaking at 37°C in thermoshaker overnight
**3**	Centrifuge at 19,980 × g for 5 min.
**4**	Supernatant + 180 µL NaCl (5 M), shake strongly 50x
**5**	Centrifuge at 19,980 × g for 5 min.
**6**	Supernatant + 420 µL Isopropanol (-20°C), shake gently 20x
**7**	Centrifuge at 19,980 × g for 5 min
**8**	Discard supernatant, + 250 µL Ethanol (80%), shake strongly 50x
**9**	Centrifuge at 19,980 × g for 5 min
**10**	Repeat step 8-9
**11**	Dry until ethanol is completely evaporated
**12**	Dilute in 50 µL demineralized H_2O

Table S1: Primers used for amplification of *Stylops* genes.

Amplified gene (region)	Primer name	Primer sequence (5'-3')	Source
COI	CO122For	TCWACAAATCATAAAATAATTGG	Jůzová et al. 2015
COI	CO1669Rev	TCCTCCTCCTAAAGGRTCRAA	Jůzová et al. 2015
H3	H3F	ATGGCTCGTACCAAGCAGACVGC	Colgan et al., 1998
H3	H3R	ATATCCTTRGGCATRATRGTGAC	Colgan et al. 1998
18S	18Sa-F	ATTAAAGTTGTTGCGGTT	Whiting et al., 1997
18S	18Sb-R	GAGTCTCGTTCGTTATCGGA	Whiting et al., 1997

References:

Colgan DJ, McLauchlan A, Wilson GDF, Livingston SP, Edgecombe GD, Macaranas J et al. (1998) Histone H3 and U2 snRNA DNA sequences and arthropod molecular evolution. *Australian Journal of Zoology*, 46, 419-437. https://doi.org/10.1071/ZO98048

Jůzová K, Nakase Y, Straka J (2015) Host specialization and species diversity in the genus *Stylops* (Strepsiptera: Stylopidae), revealed by molecular phylogenetic analysis. *Zoological Journal of the Linnean Society*, 174, 228-243. https://doi.org/10.1111/zoj.12233

Whiting MF, Carpenter JC, Wheeler QD, Wheeler WC (1997) The Strepsiptera problem: Phylogeny of the holometabolous insect orders inferred from 18S and 28S ribosomal DNA sequences and morphology. *Systematic biology,* 46, 1-68. https://doi.org/10.1093/sysbio/46.1.1

Table S5: Components for a single PCR reaction. * depending on DNA concentration

Ingredient	Volume (µL)
5x GoTaq buffer (Promega)	2.5
dNTPs (10 mM)	0.25
Forward Primer (10 µM)	0.3
Reverse Primer (10 µM)	0.3
GoTaq Polymerase (Promega) (5 u/mL)	0.1
Milli-Q® - H_2O	7.05 - 8.05 *
DNA-extract	1 - 2 *

Table S6: Used PCR cycler programs. * depending on DNA concentration

Gene	Temperature (°C)	Time (min)	
	94	02:00	
	94	00:45	
COI	50	00:45	30-35 x *
	72	01:00	
	72	05:00	
	95	02:00	
	95	01:00	
H3 / 18S	52.6	01:00	35 x
	72	01:00	
	72	05:00	

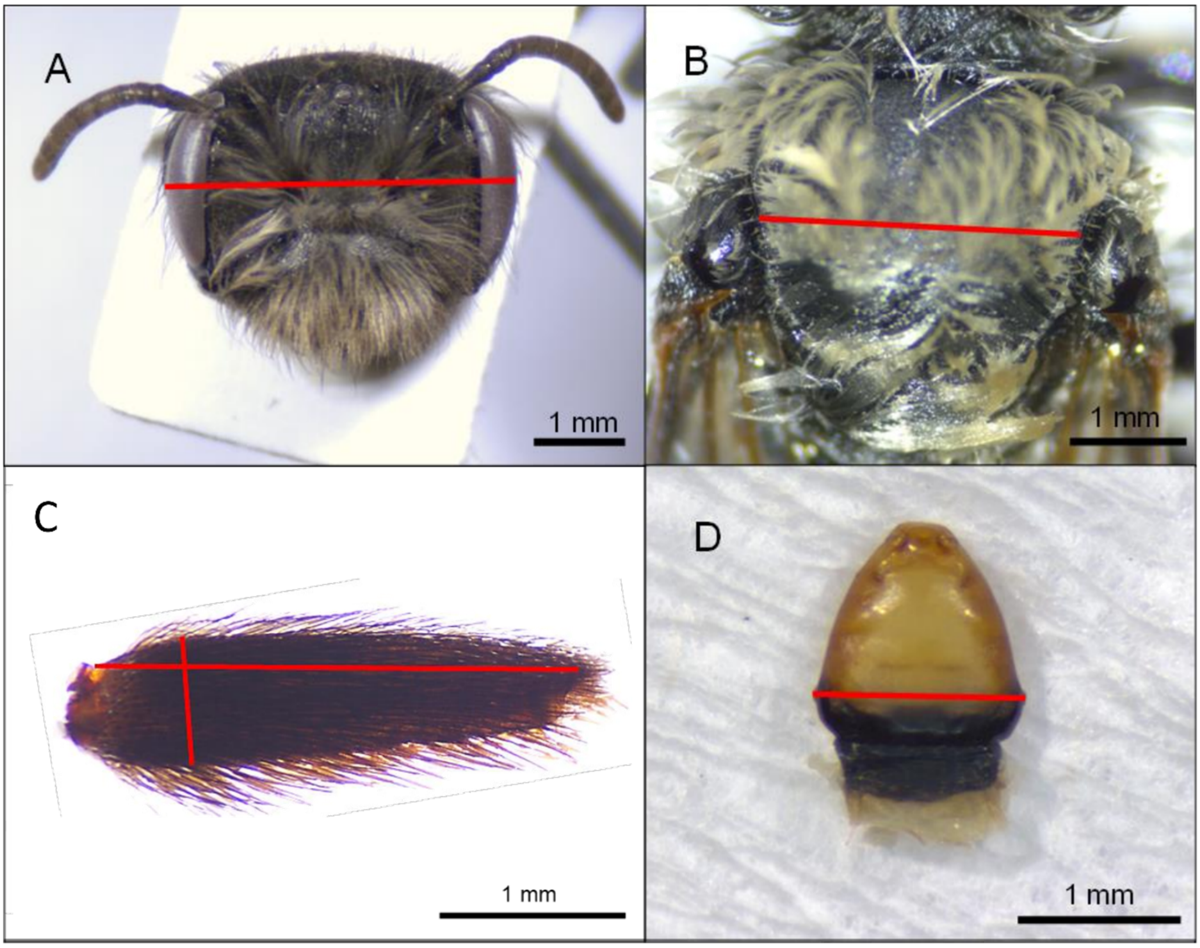

Figure S2: Morphological measurements taken on *A. vaga* and *S. ater*. (A) head width of *Andrena*, (B) intertegular distance of *Andrena*, (C) metabasitarsus length and width of *Andrena*, (D) Cephalothorax width of *Stylops*.

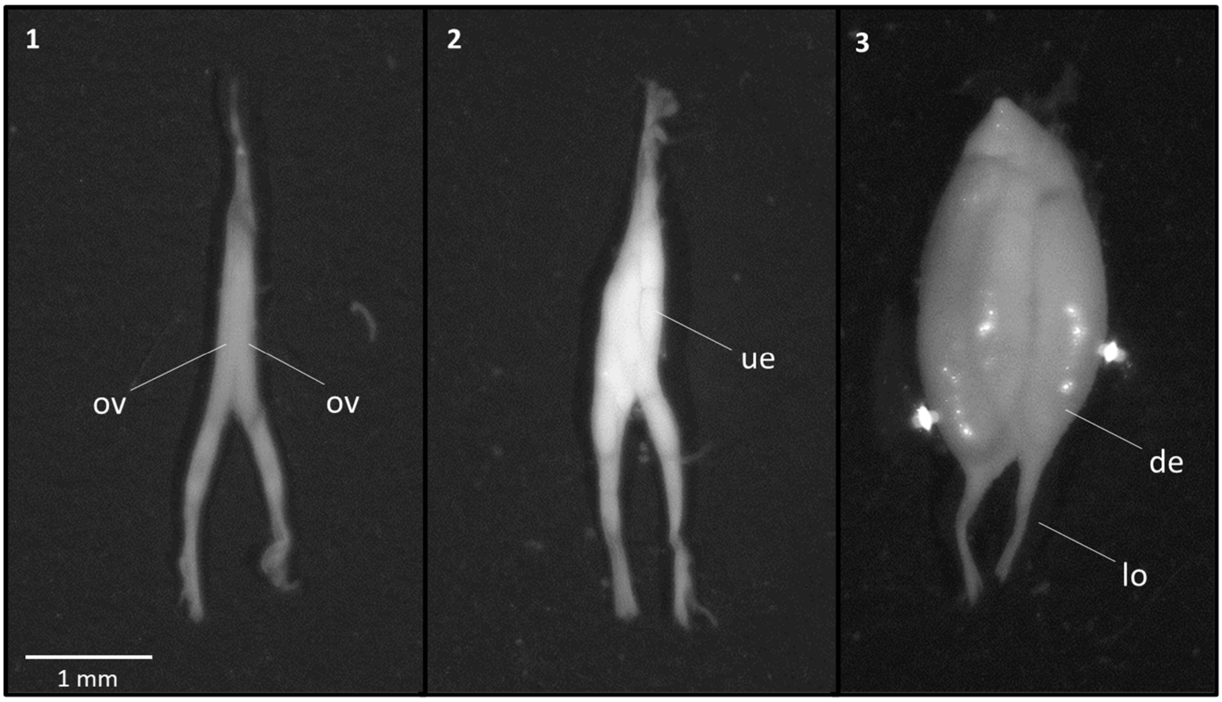

Figure S3: Ovary scores of *A. vaga* females. 1 - no clear egg development, ovaries thread-like; 2 - only small, underdeveloped eggs; 3 - large eggs of at least 1000 µm in the distal part of the ovaries (de - developed egg, lo - lateral oviduct, ov - ovary, ue - underdeveloped egg).

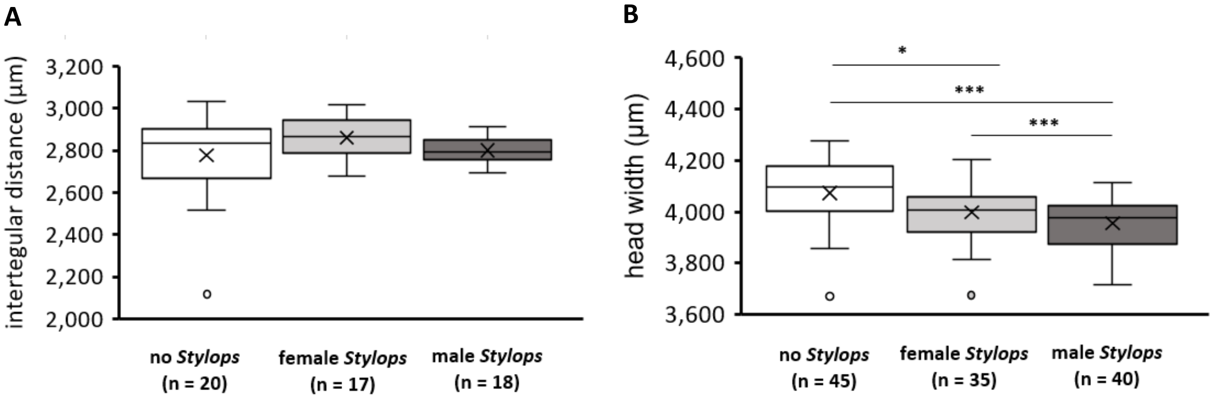

Figure S4: (A) intertegular distance of *A. vaga* females of different stylopization status (sites TU and RK), (B) head width of *A. vaga* females of different stylopization status (alternative model: all sites except MS pooled). Boxplots show minima and maxima (whiskers), medians and first and third quartiles (boxes), means (cross) and outliers (empty circles) (* $p < 0.05$, *** $p < 0.001$).

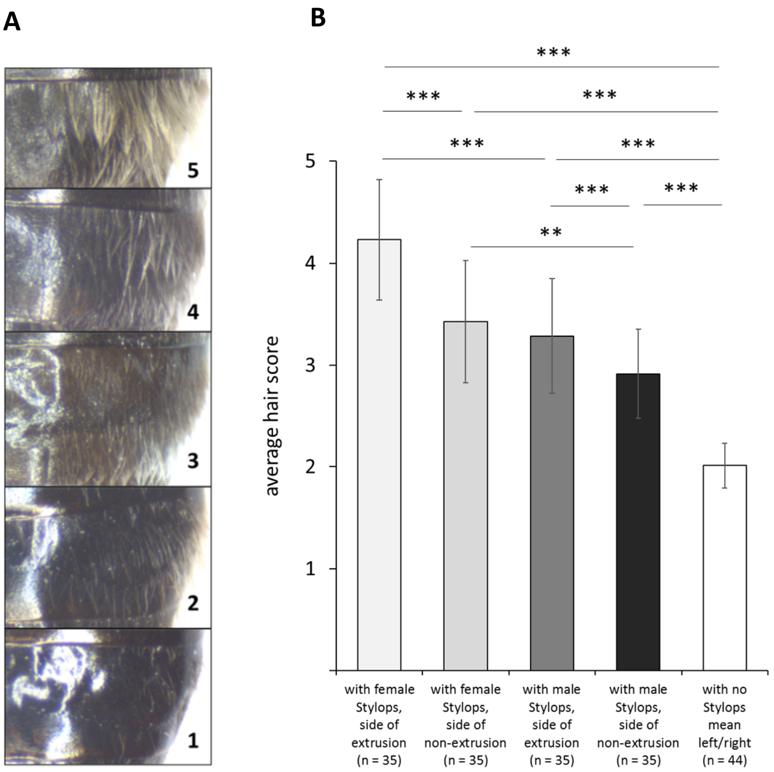

Figure S5: Hairiness of tergite 4 in *A. vaga* females of different parasitation groups. (A) Hair scores: 1 - almost no hair, 2 - sparse and short hair, 3 - partly dense and short hair, 4 - dense and partly long hair, 5 - very dense and long hair. (B) Average hair score by different parasitation types and position, error bars show standard deviations (**p < 0.01, *** p < 0.001).
